# Putative Role of Adipose Tissue in Growth and Metabolism of Colon Cancer Cells

**DOI:** 10.3389/fonc.2014.00164

**Published:** 2014-06-26

**Authors:** Betty Schwartz, Einav Yehuda-Shnaidman

**Affiliations:** ^1^Institute of Biochemistry, Food Science and Nutrition, The Robert H. Smith Faculty of Agriculture, Food and Environment, The Hebrew University of Jerusalem, Rehovot, Israel

**Keywords:** adipocytokine, colorectal cancer, leptin, mitochondrial dysfunction, Warburg effect

## Abstract

Newly emerging data highlight obesity as an important risk factor for developing certain types of cancer, including colorectal cancer. Although evidence supports a link between the two, the mechanisms responsible for this relationship have not yet been fully elucidated. Hypertrophied and dysfunctional adipose tissue of the obese state is characterized by low-grade inflammation. Adipokines and cytokines secreted from adipocytes, together with the abundant availability of lipids from adipocytes in the tumor microenvironment, promote adhesion, migration, and invasion of tumor cells and support tumor progression and uncontrolled growth. One of the predisposed targets of the deleterious effects exerted by secretions from adipose tissue in obesity is the activities associated with the cellular mitochondria. Mitochondrial oxidative metabolism plays a key role in meeting cells’ energetic demands by oxidative phosphorylation (OxPhos). Here we discuss: (a) the dynamic relationship between glycolysis, the tricarboxylic acid cycle, and OxPhos; (b) the evidence for impaired OxPhos (i.e., mitochondrial dysfunction) in colon cancer; (c) the mechanisms by which mitochondrial dysfunction can predispose to cancer. We propose that impaired OxPhos increases susceptibility to colon cancer since OxPhos is sensitive to a large number of factors that are intrinsic to the host (e.g., inflammation). Given that adipocytes are a major source of adipokines and energy for the cancer cell, understanding the mechanisms of metabolic symbiosis between cancer cells and adipocytes should reveal new therapeutic possibilities.

## Obesity and Colorectal Cancer

Cancer is one of the leading causes of death in the developed world. Colorectal cancer (CRC) is the fourth cause of cancer death, being the second most common type of cancer in women and the third most common in men (1). One of the relatively newly discussed risk factors for CRC is obesity, whose incidence is also accelerating worldwide (2).

The global obesity epidemic is thought to directly affect the incidence of CRC, with a 7% increase in CRC risk for every 2.4 unit increase in body-mass index (BMI) (3). Current epidemiological studies demonstrate that overweight and obesity can account for 14% of all cancer-related deaths in men, 20% in women (4). The results of a meta-analysis also revealed a 5% increased risk of CRC per inch of waist circumference, an indication of abdominal fat (5). Due to the excess availability of energy-dense food and physical inactivity, obesity levels have risen dramatically in western countries, including the USA (6). Indeed, the prevalence of obesity, defined by a BMI above 30 kg/m^2^, has now reached 35% with no indication of a decline (6).

The increasing prevalence of obesity encourages the performance of studies aimed at acquiring an in-depth understanding of the relationship between obesity and cancer. Identifying the mechanisms that contribute to the causal association between obesity and cancer may facilitate a better understanding of this relationship (2). Whereas there is an abundance of data supporting a link between obesity and CRC risk, the mechanisms responsible for this relationship have not yet been fully elucidated. It is critical to first understand the molecular mechanisms orchestrating the effects of obesity on CRC, with the aim of developing more effective therapeutic strategies to combat obesity-associated CRC.

This review summarizes the most recent knowledge of the mechanisms associated with the cross-reaction between adipose tissue and the colonic cell. It will try to explain why colonic cells are more susceptible to developing tumors in an obese environment (7). To do so, it is imperative to understand the normal colonic microenvironment in both experimental and human models, and then elucidate the role played by obesity in cell–cell crosstalk and delineate the underlying factors contributing to increased CRC risk.

## Adipose Tissue Physiology in Non-Obese Individuals and Its Pathology in Obesity

Adipose tissue has conventionally been regarded as an insulating and mechanically supportive tissue serving mainly for energy storage. Following the discovery of leptin in 1994, adipose tissue was considered a fully functioning endocrine organ, capable of regulating systemic energy and metabolic homeostasis. The active adipose tissue endocrine organ secretes fatty acids and peptide hormones or cytokines, collectively termed adipocytokines, which are directly involved not only in the regulation of whole-body metabolism but also in inflammatory and immune responses (8). These biologically active factors act locally or systemically to influence multiple processes, including: glucose and fatty acid metabolism, insulin sensitivity, adipocyte differentiation, inflammation, and the immune response. Adipokines are known to mediate metabolism, chemotaxis, growth, vascularization, coagulation, and inflammation (8).

The adipose tissue is composed mainly of adipocytes, but also includes a vascular portion made up of endothelial cells, pericytes, monocytes, macrophages, and pluripotent stem cells (9, 10). The anatomical location of the adipose tissue confers specific metabolic functions (11). Human omental tissue (visceral adipose tissue, VAT) expresses higher levels of proteins involved in lipid and glucose metabolism than subcutaneous adipose tissue (SAT) depots (12). Differences in gene expression and lipolytic activity have also been reported in SAT from the abdomen versus hip anatomical regions (13). There are notable differences between the amounts of SAT and VAT present in the abdominal cavity in obese versus lean subjects. Compared with SAT, VAT is more cellular, vascular, and innervated, it contains a larger number of inflammatory and immune cells, lower preadipocyte-differentiation capacity, and a greater percentage of large adipocytes (14).

Obesity is characterized by excess whole-body energy, which accumulates as triacylglycerol in the adipocytes. Obesity, particularly visceral obesity (excess VAT), is considered one of the major risk factors for developing metabolic diseases, including type 2 diabetes, cardiovascular disease, and cancer (15). Due to the significant endocrine function of the adipose tissue, numerous pathologies result from the dysfunctional adipocytes in obesity. For example, the excess fatty acids produced by dysfunctional adipocytes disrupt the cell membranes of adjacent cells, resulting in endoplasmic reticulum stress and mitochondrial damage (16). Obesity and metabolic syndrome are characterized by chronic energy surplus that ultimately induces acute deterioration of the adipose tissue’s endocrine function. Adipocytes undergo hypertrophy, and secrete increasing amounts of proinflammatory adipokines, including: monocyte chemoattractant protein (MCP)-1, tumor necrosis factor (TNF)-α, interleukin (IL)-6, IL-8, plasminogen activator inhibitor (PAI)-1, and leptin (17). This enhanced secretion of a wide variety of proinflammatory and inflammatory peptides promotes massive penetration of inflammatory cells such as macrophages, lymphocytes, and others, all of which induce significant alterations of the microenvironment within the adipose tissue. Cumulatively, these events may ultimately lead to a major change in the cellular distribution of the adipose tissue. While the inflammatory cell population may be present at up to 5–10% of total cells in the lean subjects, in the obese subjects it may reach up to 50% of the total cellular content of the adipose tissue (18). The substantial infiltration of inflammatory cells into the adipose tissue of obese subjects induces a stage of chronic inflammation that not only modifies the local metabolism, but also influences systemic energy homeostasis (19). Most of the inflammatory cells in the obese individual’s adipose tissue are activated macrophages that release a wide variety of proinflammatory cytokines such as MCP-1, TNF-α, IL-6, IL-8, and others. These secreted cytokines induce lipolysis in adipocytes while reducing their ability to synthesize triacylglycerols; this induces an elevation in circulating free fatty acids, ultimately impinging on systemic metabolic homeostasis. These liberated fatty acids may have deleterious effects in many cells, including colonic cells (20).

## Adipose Tissue and Adipocytes in Obese Individuals Support Tumorigenesis

The role of the adipose tissue in tumor initiation, growth, and metastasis is considered to be a relatively new area of investigation. Basic cancer research has traditionally focused on understanding the contribution of alterations in cancerous epithelial cells. However, the characteristics of the tissue’s proximal and distal microenvironments are proposed to play an integral role in supporting the proliferation of cancer cells (21, 22). Therefore, it is important to understand the complex mutual relationships between colon cancer cells and adipocytes and how these interactions may alter colon cancer metabolism and promote carcinogenesis.

As already noted, adipose tissue in the obese state is characterized by chronic inflammation and enriched proportions of inflammatory cells such as lymphocytes, macrophages, and stromal cells. These inflammatory cells, together with the altered resident adipocytes, secrete significant amounts of adipokines and other cytokines, which have been implicated in the promotion of tumor growth (17). Most of the adipokines secreted by the obese adipose tissue, including TNF-α, IL-6, IL-8, and MCP-1, among others, have been implicated in tumor progression (23). Inflammatory cells in obese adipose tissue are able to produce reactive oxygen species (ROS). ROS have been shown to induce mitogenic activity at low concentrations and they are therefore considered to be tumor-promoting signaling molecules (24). Chronically higher levels of both proinflammatory cytokines and ROS in obesity may promote tumorigenesis. Although it is not exactly clear how the inflammatory state in the adipose tissue is initiated, one proposed factor is hypoxia (25). During weight gain and adipose tissue expansion, some of the adipose tissue cells are pushed far from the tissue’s blood vessels, causing these cells to become poorly oxygenated, resulting in localized hypoxia. Hypoxia is known to activate hypoxia-inducible factor (HIF)-1α, which in turn induces the infiltration of macrophages and monocytes into the adipose tissue and thus upregulates the secretion of TNF-α (26). TNF-α has been shown to support cancer cell proliferation, angiogenesis, and metastasis (27, 28). One of the canonical proposed mechanisms of TNF-α-induced carcinogenesis is through activation of the nuclear transcription factor NF-κB by inhibiting the inhibitor of NF-κB (IκB) (29). Activated NF-κB has been shown to prevent apoptosis and support inflammation-associated cancers (30). Moreover, both TNF-α and hypoxic conditions induce secretion of the proinflammatory cytokine IL-6. IL-6 levels are elevated in obesity and positively correlate with BMI (31), and a role for IL-6 in tumorigenesis has been demonstrated in IL-6-deficient animal models that do not develop tumors (32). The enhanced secretion of inflammatory cytokines during obesity and the correlation of the inflammatory stage to carcinogenesis clearly correlate the elevated cytokine levels generated during obesity to advanced carcinogenesis.

Aside from cytokine secretion, the adipose tissue is the major endocrine organ synthesizing and secreting adipokines. Adipokines are hormones derived from adipocytes that play a key role in energy homeostasis. Obesity not only alters cytokine secretion but also adipocytokine secretion. As already mentioned, some adipocytokines such as TNF-α induce increased angiogenesis, with angiogenesis being one of the key steps involved in the development of certain types of cancer, including CRC (33, 34). Among the adipocyte-derived cytokines, the serum level of leptin is closely related to the amount of adipose tissue in humans (35). Leptin informs the brain of the body’s energy status through activation of the leptin receptor and causes adjustments in food intake (35). Obesity is associated with alterations in leptin regulation; chronic overexpression of leptin induces leptin resistance, resulting in enhanced levels of circulating leptin. This is similar to the increased insulin levels seen in insulin resistance, which is also associated with increased adiposity (36). The close relationship between adiposity and leptin levels suggests that this hormone plays a role in the increased incidence of obesity-associated cancer. Excessive expression of leptin and/or leptin receptor has been reported as a risk factor for cancer (37). It has been proposed that obesity-related cancers are due, in part, to changes in the levels of adipokines secreted by adipocytes, infiltrating macrophages, or associated stromal cells. Although numerous adipokines have been identified, only a few have been extensively studied for their involvement in promoting or inhibiting colon cancer tumor growth. These include leptin, resistin, adiponectin, and others (38). Elevated levels of circulating leptin have been shown to increase the risk of colon cancer (37) and other malignancies. Elevated leptin in cancer has been suggested to have several protumorigenic effects. Leptin shows mitogenic activity in cancers of the colon (39) and has mitogenic and antiapoptotic effects (40). A previous study conducted in our laboratory demonstrated that leptin affects processes related to colon cancer initiation and progression *in vitro* (41). Taken together, the effects of elevated leptin in obesity can drive CRC tumor growth and progression.

## Metabolic Reprograming and CRC

Metabolic reprograming is a common feature in most cancers. Over 90 years ago, Otto Warburg suggested that during cancer development, glycolysis increases (42, 43). Warburg proposed that the metabolic shift toward glycolysis is due to injury of the oxidative phosphorylation (OxPhos) system. In contrast to normal cells, which rely primarily on mitochondrial OxPhos for ATP production, most cancer cells rely more heavily on aerobic glycolysis, a phenomenon termed “the Warburg effect” (42, 43). These observations provided the basis for developing the [18F]2-fluoro-2′-deoxy-d-glucose-positron emission tomography (FDG-PET) technique used today for cancer examinations (44). Based on the Warburg effect, it is predicted that if cells rely more heavily on aerobic glycolysis, mitochondrial OxPhos will become dysfunctional. Integrity of the OxPhos system is critical for optimal energy production, but optimal assembly of the OxPhos system is a highly complex process. Impairments of the OxPhos system are associated with respective modifications of cellular redox status and enhanced production of ROS. Indeed, a large number of studies indicate that the mitochondria play a key role during tumorigenesis [reviewed in Ref. (45)], where they can be dysfunctional due to defects in the OxPhos process, lower levels of mitochondrial DNA (mtDNA). Some studies have analyzed mutations in mtDNA, which is relatively more susceptible to damage than nuclear DNA (nDNA). It has been estimated that nearly 70% of human CRCs contain mtDNA mutations (46), and these mutations have been found in several mitochondrial complex-associated genes. For example, NDUFA13 (GRIM-19), an essential subunit of complex I, is downregulated in colon carcinogenesis (47); mutations in SDHs–complex II subunits, lead to tumorigenesis, and they are therefore considered to be tumor-suppressor genes; similarly, the expression level of COX2–complex IV subunit 2, is reduced in many types of cancers (48). Almost complete loss of complex I (mostly due to mtDNA mutations) is associated with some tumors, such as in breast cancer (49). Mutations in subunits NDUFA4 and NDUFA5 affect complex I function and the metastatic properties of the breast cancer cell line MDA-MB-231 (50). Downregulated expression of the β-subunit of complex V (β-F1-ATPase) in cancer is associated with a respective increase in glyceraldehyde-3-phosphate dehydrogenase activity. Therefore, measurement of the relative expressions of β-F1-ATPase and glyceraldehyde-3-phosphate dehydrogenase may have prognostic value for breast and other cancers (51). Moreover, mtDNA is reduced in cancer and impaired mitochondrial metabolism may also play a key role in cancer development (52, 53). The mechanism may include increased mitochondrial degradation (mitophagy), lower mitochondrial proliferation, or both (52). Ussakli et al. (54) recently reported that mitochondrial loss precedes the development of dysplasia, and that it might be useful in detecting, and potentially predicting cancer. Cook and Higuchi (53); Cook and Higuchi (53) recently demonstrated that a decrease in mtDNA is correlated with the progression and aggressiveness of tumor growth. This phenomenon facilitates the use of mtDNA as a diagnostic tool for evaluating cancer progression. The decreased expression of mitochondrial genes may be due to the enhanced oxidative stress conferred by cancer cells (55).

To date, most studies have focused on the effects of tumor suppressors and oncogenes on cellular metabolism to explain the Warburg effect, with the main assumption that metabolic alterations are a consequence of transformation (56). In line with this assumption, oncogenes were shown to favor glycolysis while tumor suppressors were associated with induction of oxidative metabolism (57). Taken together, alterations in the activities of tumor suppressors and oncogenes during carcinogenesis induce metabolism reprograming in the transformed cells to supply their metabolic needs (58). These findings evolved into the hypothesis that altered metabolism plays a primary role in cancer development, rather than being only a marginal event (59). Mutations in p53 and mtDNA are detected in most cancers, supporting the notion that mitochondrial dysfunction and cancer development are closely related (60). For example, mitochondrial dysfunction may promote cancer development by: (i) activation of AKT and HIF1α pathways (61, 62), (ii) suppression of p53 and PTEN function, (iii) induction of resistance to cell death (63), and (iv) a metabolic shift toward glycolysis (64). Supporting evidence for this hypothesis was obtained from assessing the expression of glycolytic enzymes such as pyruvate kinase (PKM2) and isocitrate dehydrogenase (IDH1, 2), among others, and demonstrating that they enhance cellular transformation.

## Metabolic Reprograming in Obesity-Associated CRC

Metabolic reprograming has been found to be altered in obesity (65), and in cancer (66). The question is whether obesity impinges directly on epithelial cell metabolism and induces the development of different types of cancer, particularly those of peripheral tissues that are near the VAT (e.g., liver, colon, pancreas, and kidney).

A reduction in mtDNA has been found in adipocytes from obese individuals (65). That study demonstrated that mitochondria in white adipose tissue play an important role in energy metabolism and in the development of obesity, insulin resistance and, ultimately, type 2 diabetes. Supporting this view, Wilson-Fritch et al. (67) demonstrated that fat cells from ob/ob mice display reduced mitochondrial mass and altered mitochondrial structure, which could be normalized by treatment with the PPAR-γ agonist rosiglitazone. Lindinger et al. (68) evaluated the mtDNA content per cell of omental adipose tissue and found that it tended to be lower in patients with diabetes, which may partly explain the impairment of mitochondrial function observed in insulin resistance. The number of mitochondria in white adipocytes is relatively low. The biogenesis of mitochondria and, thus, their numbers, are controlled by different extracellular and intracellular stimuli, a case in point being changes in temperature (69). Cellular mitochondrial content is also controlled by fission and fusion processes (70). In cases of mitochondrial stress, mitochondria can divide or fuse to restore their function. During the fusion process, mitochondria distribute mitochondrial proteins and exchange mtDNA; fusion is followed by a fission process, which leads to newly “renovated” mitochondria. This fusion–fission process may be dysregulated in obesity (71). Taken together, these findings support the idea that the mitochondria in adipose tissue play an important role in energy metabolism and in the development of obesity and associated diseases.

Mitochondrion-derived proteins, such as NDUFA4L2 (a complex I inhibitor) and the deacetylase SIRT3, among others, are associated with both carcinogenesis and cellular respiration (72–74). OxPhos deficiency has been shown to suppress p53 expression/function in several cell types (75). Mitochondrial dysfunction caused by extensive mtDNA mutations is suggested to play a key role in aging and other diseases, including cancer (66). These findings therefore suggest that declining OxPhos function could play a causative role in increasing cancer incidence with age.

A link between obesity, inflammation, metabolic reprograming, and cancer was addressed in a study in which chronic inflammation associated with obesity was shown to facilitate cancer initiation and progression by suppressing OxPhos (76, 77). Garcia-Ruiz et al. (78) showed that inflammation and oxidative stress induced by TNF-α are associated with damage to the OxPhos system in liver tissue of ob/ob mice. Inflammation can occur through localized secretion of factors such as TNF-α, Wnt peptides, leptin, etc., all of which can impair OxPhos activity (79). Yadava et al. (66) showed that mitochondrial complexes I, III, and IV are affected by inflammatory cytokines. These authors demonstrated that low concentrations of TNF-α (too low to induce cell death) significantly reduce the expression levels of the NDUFS3 subunit of complex I in HEK293 cells. Similarly, treatment of primary human mammary epithelial cells with TNF-α induced a significant reduction in mitochondrial complex I-associated respiration, which was already detectable within 1 h of exposure to TNF-α. Wnt proteins can promote the epithelial-to-mesenchymal transition (EMT) and stem cell-like behavior of colonic epithelial cells (80). Dickkopf-1 (Dkk1) is a potent inhibitor of Wnt signaling, and the expression of Dkk1 was reduced in colon cancer, with a concomitant reduction in immunohistochemical features of EMT (such as increased expression of the epithelial marker E-cadherin, decreased expression of the mesenchymal marker vimentin, and cytoplasmic distribution of β-catenin). Furthermore, Dkk1 overexpression resulted in restoration of the epithelial phenotype and decreased expression of EMT transcription factors Snail and Twist, and decreased the expression of markers of intestinal stem cells such as cluster of differentiation 133 (CD133) and leucine-rich-repeat-containing G-protein-coupled receptor 5 (Lgr5). Interestingly, exposure of cancer cells to Wnt proteins has been shown to inhibit OxPhos as well, and to enhance glycolysis through the downregulation of complex IV subunits (81). Therefore, inflammation mediated by TNF-α or Wnt proteins may influence cancer progression though alterations of mitochondrial metabolism.

**Figure 1:**
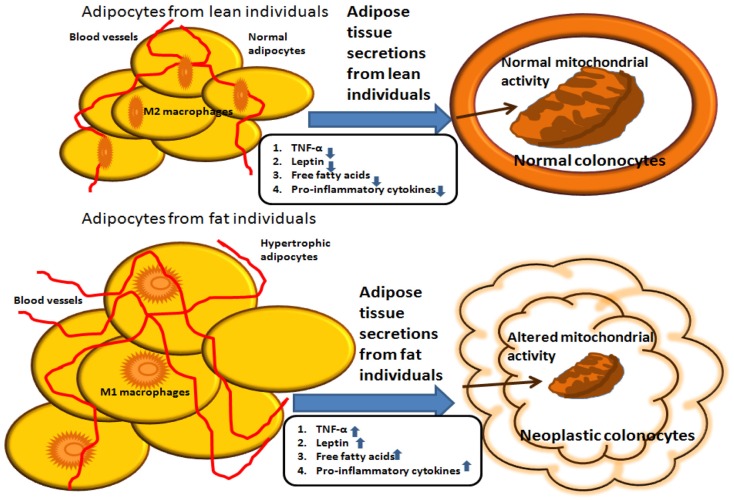
**Summary of potential interactions between adipocytes from lean versus obese individuals**. Molecules secreted from the adipose tissue of obese individuals induce mitochondrial dysfunction, which is associated with carcinogenesis. In contrast, molecules secreted from the adipose tissue of lean individuals do not alter mitochondrial metabolic activity in colonocytes.

The effect of adipokines on mitochondrial activity may provide a link via which adipose tissue secretions can influence the outcome of colon cancer (see Figure 1). We recently addressed the issue of metabolic reprograming in obesity-associated CRC and demonstrated that secreted products from the adipose tissue of obese subjects inhibit mitochondrial respiration and function in HCT116 colon cancer cells, an effect that is at least partly mediated by leptin (82). As already mentioned, leptin is an adipokine that is synthesized and secreted by the adipose tissue, and its systemic levels mirror fat adiposity. Leptin is predominantly involved in the regulation of food intake and energy homeostasis via central activities (83). In addition, leptin has been shown to exert a wide variety of effects peripherally, activities associated with the immune system, angiogenesis, interaction with signaling pathways of growth hormones, and lipid metabolism. A vast number of reports suggest that leptin is a legitimate key candidate linking obesity to carcinogenesis (37, 39, 41, 84). A previous study conducted in our laboratory supports the view that leptin may directly affect processes related to colon cancer initiation and progression in colon cancer cells *in vitro* (41). Furthermore, in our recent study (82), we demonstrate that colon cancer cells exposed to conditioned media prepared from VAT from obese subjects express lower oxygen consumption rate (OCR) levels than colon cancer cells exposed to conditioned media obtained from VAT from lean subjects. The lower OCR levels were associated with lower expression levels of nuclear- and mitochondrion-encoded genes, suggesting a central role for mitochondria in the metabolic reprograming of colon cancer by obesity.

## Concluding Remarks

Taken together, the reviewed studies suggest that one of the most important links between adipose tissue secretions from obese individuals and colon cancer progression is the mitochondrial dysfunction induced by adipocyte secretions in colonic cells. From our recent study, we are able to pinpoint the adipokine leptin as one of the direct links between adipose tissue secretions from obese subjects. The exact molecular signaling pathways mediating this effect have yet to be discovered; however, we have previously found that leptin promotes the metastatic potential of colon cancer cells by affecting PI3K and Src kinase pathways and activating Rac1 and Cdc42 (41).

Understanding the molecular mechanisms by which obesity increases colon cancer risk will help in designing novel strategies to prevent the increasing number of cases of obesity-related colon cancer.

## Conflict of Interest Statement

The authors declare that the research was conducted in the absence of any commercial or financial relationships that could be construed as a potential conflict of interest.
